# Bilateral optic neuropathy following prolonged linezolid use: A case report

**DOI:** 10.1016/j.idcr.2026.e02524

**Published:** 2026-02-19

**Authors:** Hadiza Ometere Ibrahim, Aaesha Easa Alnuaimi, Amani Alzaabi, Mahfoud Elbashari, Mohammed Abouelnaga, Amna Almaazmi

**Affiliations:** a Department of Internal Medicine, Zayed Military Hospital, Abu Dhabi, United Arab Emirates; b Department of Ophthalmology, Zayed Military Hospital, Abu Dhabi, United Arab Emirates; c Department of Neurology, Zayed Military Hospital, Abu Dhabi, United Arab Emirates

**Keywords:** Linezolid, Optic neuropathy, Visual loss, Drug toxicity, Mitochondrial toxicity, Optical coherence tomography

## Abstract

This report describes a case of bilateral linezolid-associated optic neuropathy in a 65-year-old woman treated for *Mycobacterium abscessus* infection and highlights the importance of early recognition and intervention. The patient developed progressive, painless bilateral visual decline after more than six months of linezolid therapy. Ophthalmic examination revealed optic disc edema (left greater than right), impaired color vision, and prolonged visual evoked potential latencies. Neuroimaging excluded compressive or inflammatory etiologies, while optical coherence tomography demonstrated bilateral retinal nerve fiber layer thickening, with subsequent imaging showing optic atrophy in the left eye. Linezolid was discontinued, and a short empirical course of corticosteroids was administered. Visual function partially improved in the left eye following cataract extraction; however, residual optic atrophy and dyschromatopsia persisted. This case reinforces that linezolid-associated optic neuropathy is a potentially reversible yet under-recognized complication of prolonged therapy and emphasizes the need for routine ophthalmologic surveillance in patients receiving linezolid beyond recommended durations. A high index of suspicion is essential, as progressive bilateral visual loss may be misattributed to common ocular comorbidities such as cataracts, leading to delayed diagnosis and irreversible visual impairment.

## Highlights

• Bilateral optic neuropathy occurred after prolonged linezolid use.
• OCT showed RNFL thickening and later optic atrophy.
• Visual symptoms partially improved after drug cessation and cataract surgery.
• Peripheral neuropathy supported mitochondrial toxicity.
• Early ophthalmologic monitoring is essential in long-term linezolid therapy.

## Introduction

Linezolid is an oxazolidinone-class antibiotic widely used in the treatment of multidrug-resistant gram-positive infections. Its antimicrobial activity is mediated through inhibition of the 23S rRNA component of the bacterial 50S ribosomal subunit, thereby impairing protein synthesis [1]. Owing to structural similarities between bacterial and mitochondrial ribosomes, linezolid may also interfere with mitochondrial protein synthesis, resulting in cellular energy dysfunction and toxicity in metabolically active tissues such as the optic nerve [2], [3].

Although generally well tolerated during short-term therapy, prolonged linezolid exposure has been associated with serious adverse effects, including myelosuppression, lactic acidosis, peripheral neuropathy, and optic neuropathy [4], [5]. Linezolid-associated optic neuropathy is an established but uncommon complication that may develop insidiously and progress to irreversible visual impairment if not recognized promptly [6], [7]. Despite existing recommendations to limit therapy duration, extended treatment courses remain necessary in selected patients with complex infections, increasing the risk of cumulative toxicity [8].

In clinical practice, the diagnosis of toxic optic neuropathy may be challenging, particularly in older patients or in those with coexisting ocular comorbidities such as cataracts, which can obscure early symptoms and delay specialist referral [9]. As a result, visual decline may be incorrectly attributed to more common age-related conditions rather than medication-related toxicity.

This report presents a case of bilateral linezolid-associated optic neuropathy in a patient receiving prolonged therapy for Mycobacterium abscessus infection. The case serves as a clinical reminder of the potential for visual toxicity during extended treatment and highlights the importance of early recognition, careful monitoring, and timely drug discontinuation in real-world practice.

## Case presentation

A 65-year-old woman with a background of hypertension and well-controlled non-insulin dependent type 2 diabetes mellitus (HbA1c 6.2 %) for approximately five years, was being treated for *Mycobacterium abscessus* cavitary lung infection. Her regimen included oral linezolid 600 mg twice daily and intravenous amikacin, later switched to tigecycline due to side effects. After approximately 6.5 months of continuous linezolid therapy, the patient presented with progressive, painless bilateral visual decline, particularly affecting the left eye.

At four months into therapy, a routine ophthalmologic exam revealed best-corrected visual acuity (BCVA) of 6/12 in the right eye (OD) and 6/18 in the left eye (OS), with nuclear sclerosis grade 3 (NS3) cataracts and pseudoexfoliation in both eyes. Fundus photos showed normal optic disc margins.

Approximately 2.5 months later, the patient experienced a sudden, painless

worsening of vision, particularly in the left eye. BCVA had declined to 6/36 OS,

while OD remained 6/12, intraocular pressure 17/17 mmHg. A color vision

assessment indicated significant reduced color perception in the left eye, and while

both pupils reacted to light, the left eye showed a slightly sluggish response.

Notably, there was no relative afferent pupillary defect (RAPD) observed. A test for

red color saturation revealed decreased sensitivity in the left eye. The anterior

segment examination was largely unremarkable except for the presence of a

pseudo-exfoliation cataract as previously documented.

**Fig. 1:**
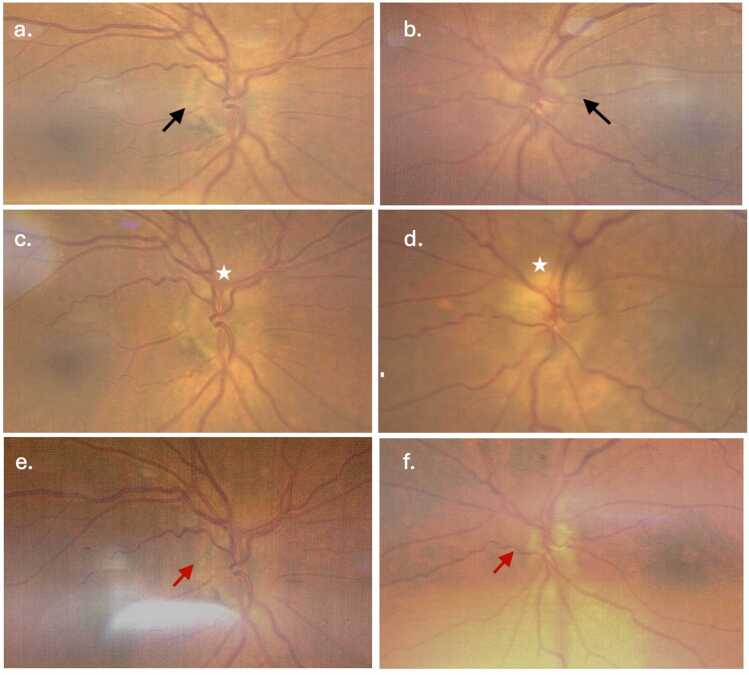
(a–b) Fundus photographs of the right and left eyes at initial presentation demonstrating mild blurring of the nasal optic disc margins (black arrows). (c–d) Images obtained at peak visual impairment showing more prominent optic disc edema, particularly along the superior margin of the left optic disc (stars). (e–f) Follow-up fundus photographs demonstrating resolution of disc edema. The right optic disc margins and color are within normal limits, while the left optic disc shows optic atrophy following resolution of prior swelling (red arrows).

A comprehensive dilated fundus examination showed bilateral blurred optic disc

margins, more prominent elevation along the margins of the left optic disc, with

hyperemia accompanied by a dull macular reflex, as illustrated in Fig. 1a-d.

**Fig. 2:**
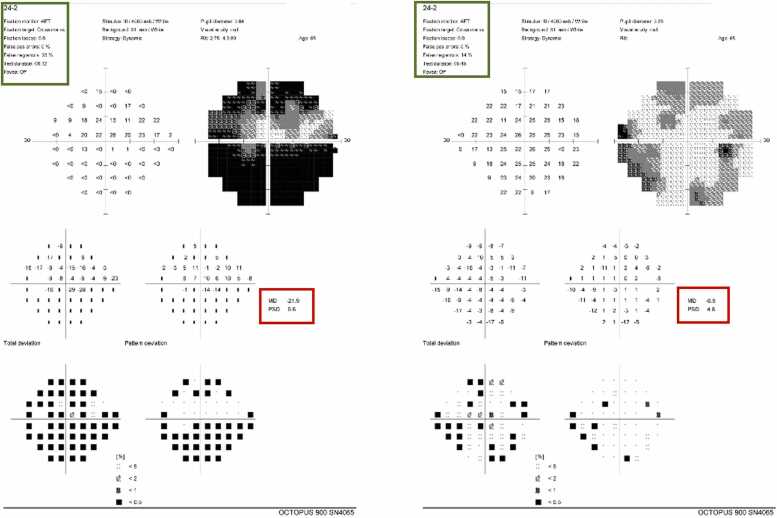
Humphrey visual field testing (24−2) of the right and left eyes. The left eye demonstrates a central visual field defect on pattern deviation analysis (red boxes), although overall test reliability was low (green boxes).

**Fig. 3:**
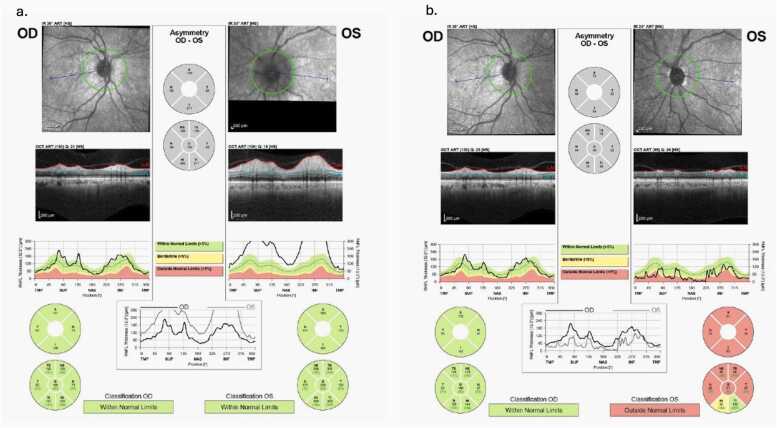
(a) Optical coherence tomography retinal nerve fiber layer (RNFL) analysis demonstrating bilateral thickening, more pronounced in the left eye, consistent with optic disc edema. (b) Follow-up RNFL analysis showing thinning of the left optic nerve, consistent with optic atrophy following resolution of edema.

Visual fields (Humphrey 24–2) showed a central scotoma in OS despite low

reliability (Fig. 2). OCT revealed increased retinal nerve fiber layer (RNFL)

thickness bilaterally (Fig. 3a). VEP testing showed prolonged P100 latency

(157 ms OS). MRI of the brain and orbits was normal except for perineural CSF

prominence. Lumbar puncture and blood work ruled out infectious, inflammatory,

and autoimmune etiologies.

The findings were consistent with linezolid-induced optic neuropathy. Linezolid was discontinued at 6.5 months. A short course of corticosteroids was given due to the presence of papillitis.

At one-month follow-up, optic disc edema had resolved bilaterally. Red color saturation in the right eye remained normal, with Ishihara color plate testing of 12/14. However, the left optic nerve developed atrophy with

consistent reduced red saturation and reduced Ishihara colour plates identification

(3/14) (Fig. 1e–f). OCT showed RNFL thinning consistent with optic atrophy

(Fig. 3b). The patient also developed peripheral neuropathy during linezolid

therapy, supporting mitochondrial toxicity. She later underwent left cataract

extraction, improving BCVA to 6/24 OS.

## Discussion

Linezolid-associated toxic optic neuropathy is an uncommon but well-recognized adverse effect that is primarily attributed to mitochondrial dysfunction [2], [3]. Owing to structural similarities between bacterial and mitochondrial ribosomes, linezolid inhibits mitochondrial protein synthesis, leading to impaired oxidative phosphorylation and reduced ATP production in metabolically active tissues such as the optic nerve [2]. This mechanism explains the susceptibility of the visual pathway to cumulative drug toxicity during prolonged exposure.

Clinically, linezolid-induced optic neuropathy typically presents with bilateral, painless, progressive visual loss, central or cecocentral scotomas, and dyschromatopsia [4], [5]. These features were evident in our patient, who demonstrated visual field defects, prolonged visual evoked potential latencies, and optical coherence tomography–confirmed optic disc edema. Neuroimaging and cerebrospinal fluid analysis excluded compressive, inflammatory, and infectious causes, supporting a toxic-metabolic etiology.

Previous studies suggest that visual recovery following linezolid cessation is variable, with complete recovery reported in approximately two-thirds of affected patients [6]. In the present case, prompt drug discontinuation likely prevented further deterioration; however, residual optic atrophy and persistent color vision deficits remained. Although cataract extraction improved visual acuity, functional deficits persisted, highlighting the potential for irreversible damage despite timely intervention.

The development of concomitant peripheral neuropathy in our patient further supports a unifying diagnosis of linezolid-induced mitochondrial toxicity affecting both central and peripheral nervous systems [3], [8]. This multisystem involvement underscores the importance of recognizing early neurologic and ophthalmic symptoms in patients receiving prolonged therapy.

Corticosteroids are not routinely recommended in the management of toxic optic neuropathies, including linezolid-associated optic neuropathy, due to the lack of evidence supporting their efficacy in this setting [9]. In our case, a short empirical course of corticosteroids was administered because of the presence of optic disc edema suggestive of possible inflammatory involvement (papillitis). However, visual stabilization was more likely attributable to drug discontinuation rather than steroid therapy. Clinicians should therefore exercise caution when considering corticosteroids and prioritize prompt cessation of the offending agent.

This case reinforces the importance of early detection and regular ophthalmologic monitoring in patients receiving linezolid beyond recommended durations, particularly when prolonged treatment is unavoidable [7]. In real-world practice, diagnosis may be delayed by coexisting ocular conditions such as cataracts, which can obscure early manifestations of optic neuropathy. Maintaining a high index of suspicion is therefore essential to prevent irreversible visual impairment.

Although this report does not present a novel mechanism of toxicity, it serves as a practical clinical reminder of the diagnostic challenges and management considerations associated with prolonged linezolid therapy. As the use of linezolid continues to expand in the treatment of resistant infections, heightened awareness and structured monitoring protocols are critical to minimizing vision-threatening complications.

## Conclusions

Linezolid-associated optic neuropathy is an uncommon but potentially vision-threatening complication of prolonged therapy. This case highlights the diagnostic challenges encountered in real-world clinical practice, particularly in patients with coexisting ocular comorbidities that may obscure early symptoms. Prompt recognition and discontinuation of linezolid remain the cornerstone of management and are essential to prevent irreversible visual impairment. Clinicians should maintain a high index of suspicion and consider regular ophthalmologic monitoring in patients receiving extended courses of linezolid. Increased awareness and early intervention are critical to minimizing long-term visual morbidity.

## CRediT authorship contribution statement

**Ibrahim Hadiza:** Writing – review & editing, Writing – original draft, Visualization, Validation, Supervision, Software, Resources, Project administration, Methodology, Investigation, Formal analysis, Data curation, Conceptualization. **Alnuaimi Aaesha:** Writing – review & editing, Writing – original draft, Investigation, Formal analysis, Data curation. **Amani Alzaabi:** Writing – review & editing, Writing – original draft, Visualization, Supervision, Resources, Project administration, Methodology, Investigation, Formal analysis, Data curation, Conceptualization. **Mahfoud Elbashari:** Writing – review & editing, Writing – original draft, Supervision, Resources, Methodology, Investigation, Formal analysis, Conceptualization. **Mohammed Abouelnaga:** Writing – review & editing, Supervision, Methodology, Formal analysis, Conceptualization. **Amna Almaazmi:** Writing – review & editing, Writing – original draft, Visualization, Supervision, Methodology, Investigation, Formal analysis, Data curation, Conceptualization.

## Patient consent

Written informed consent was obtained from the patient for publication of this case and accompanying images.

## Declaration of Competing Interest

The authors declare that they have no known competing financial interests or personal relationships that could have appeared to influence the work reported in this paper.
